# Inverted Quantum Dot Light Emitting Diodes using Polyethylenimine ethoxylated modified ZnO

**DOI:** 10.1038/srep08968

**Published:** 2015-03-10

**Authors:** Hong Hee Kim, Soohyung Park, Yeonjin Yi, Dong Ick Son, Cheolmin Park, Do Kyung Hwang, Won Kook Choi

**Affiliations:** 1 Interface Control Research Center, Future Convergence Research Division, Korea Institute of Science and Technology (KIST), Seoul 136–791, Korea; 2 Department of Materials Science and Engineering, Yonsei University, Seoul 120–749, Korea; 3 Institute of Physics and Applied Physics, Yonsei University, Seoul 120–749, , Korea; 4 Soft Innovative Materials Research Center, Korea Institute of Science and Technology (KIST), Jeonbuk 565–905, Korea; 5 Department of Nanomaterials and Nano Science, University of Science and Technology (UST), Daejun 305–350, Korea

## Abstract

Colloidal quantum dots (QDs) are an emerging class of new materials due to their unique physical properties. In particular, colloidal QD based light emitting diodes (QDLEDs) have been extensively studied and developed for the next generation displays and solid-state lighting. Among a number of approaches to improve performance of the QDLEDs, the most practical one is optimization of charge transport and charge balance in the recombination region. Here, we suggest a polyethylenimine ethoxylated (PEIE) modified ZnO nanoparticles (NPs) as electron injection and transport layer for inverted structure red CdSe-ZnS based QDLED. The PEIE surface modifier, incorporated on the top of the ZnO NPs film, facilitates the enhancement of both electron injection into the CdSe-ZnS QD emissive layer by lowering the workfunction of ZnO from 3.58 eV to 2.87 eV and charge balance on the QD emitter. As a result, this device exhibits a low turn-on voltage of 2.0–2.5 V and has maximum luminance and current efficiency values of 8600 cd/m^2^ and current efficiency of 1.53 cd/A, respectively. The same scheme with ZnO NPs/PEIE layer has also been used to successfully fabricate green, blue, and white QDLEDs.

In the past decade, colloidal quantum dots (QDs) have been a subject of active research due to their unique physical properties of size dependent energy band gap, narrow spectral emission bandwidths, broad spectral photo response from ultraviolet to infrared, and compatibility with solution process^1^,^2^,^3^,^4^,^5^,^6^. In particular, colloidal QD based light emitting diodes (QDLEDs) have attracted considerable attention as an emerging technology for next generation displays and solid-state lighting^2^,^3^,^7^,^8^,^9^. Much effort has been devoted to enhance luminous efficiency of QDLED, and as a result, its performance has been approaching that of organic light emitting diodes (OLEDs)^3^,^7^,^10^,^11^,^12^,^13^,^14^,^15^,^16^,^17^. While a number of approaches to improve performance of the device have been proposed, the most practical one is optimization of charge transport and charge balance in the recombination region by carefully selecting charge injection/transport layer and engineering interface between the QD emitter and transport layer^2^,^3^,^7^,^15^,^16^,^17^. For example, B. S. Mashford *et al*. recently reported on state-of-the-art performance of QDLED (maximum luminance over 50,000 Cd/m^2^ and current efficiency over 10 Cd/A), comparable to that of phosphorescent OLEDs, using such an approach^7^.

Polyethylenimine ethoxylated (PEIE) is a promising surface modifier to enhance electron injection in to the QD emission layer and to control interfacial property for uniform film formation of the QD layer^18^. PEIE can be easily incorporated on top of metal, transparent conducting oxide, and conducting organic materials to modulate their electronic structures by lowering the workfunctions (WFs)^19^. In addition, a PEIE layer also induces uniform surface density of QDs by coupling of aliphatic amine functional groups in PEIE with sulfur in CdSe-ZnS QD^18^. Therefore, in our previous study, we have adopted PEIE as the electron injection layer material in an inverted structure QDLED^18^. The PEIE layer substantially reduced the WF of the ITO electrode, while playing a significant role in formation of a uniformly distributed CdSe–ZnS QDs monolayer. The resulting device exhibited maximum luminance of 2900 cd/m^2^ and luminance efficiency of 0.51 cd/A, but there was still a room for further enhancement of the performance by optimizing charge balance in the QD emitter.

Here, we incorporate the PEIE layer onto a colloidal ZnO nanoparticles (NPs) film as electron injection/transport layer (EIL/ETL) on top of the ITO electrode. The ZnO NPs film has been widely used to improve electron injection and charge balance of QD emitters^7^,^16^,^17^. The PEIE surface modifier, incorporated on the top of the ZnO NPs film facilitates the enhancement of both electron injection into the CdSe-ZnS QDs emissive layer by lowering the WF of ZnO from 3.58 eV to 2.87 eV and charge balance on the emission layer. As a result, red CdSe-ZnS QDLEDs with the ZnO NPs/PEIE layer exhibit a low turn-on voltage of 2–2.5 V, with maximum luminance and current efficiency values of 8600 cd/m^2^ and current efficiency of 1.53 cd/A, respectively, at least three times higher than the previous device with PEIE single layer. Furthermore, the same scheme with the ZnO NPs/PEIE layer has been used to successfully fabricate green, blue, and white QDLEDs.

## Results and Discussion

### Inverted red QDLED fabrication and material characterizations

**Figure 1:**
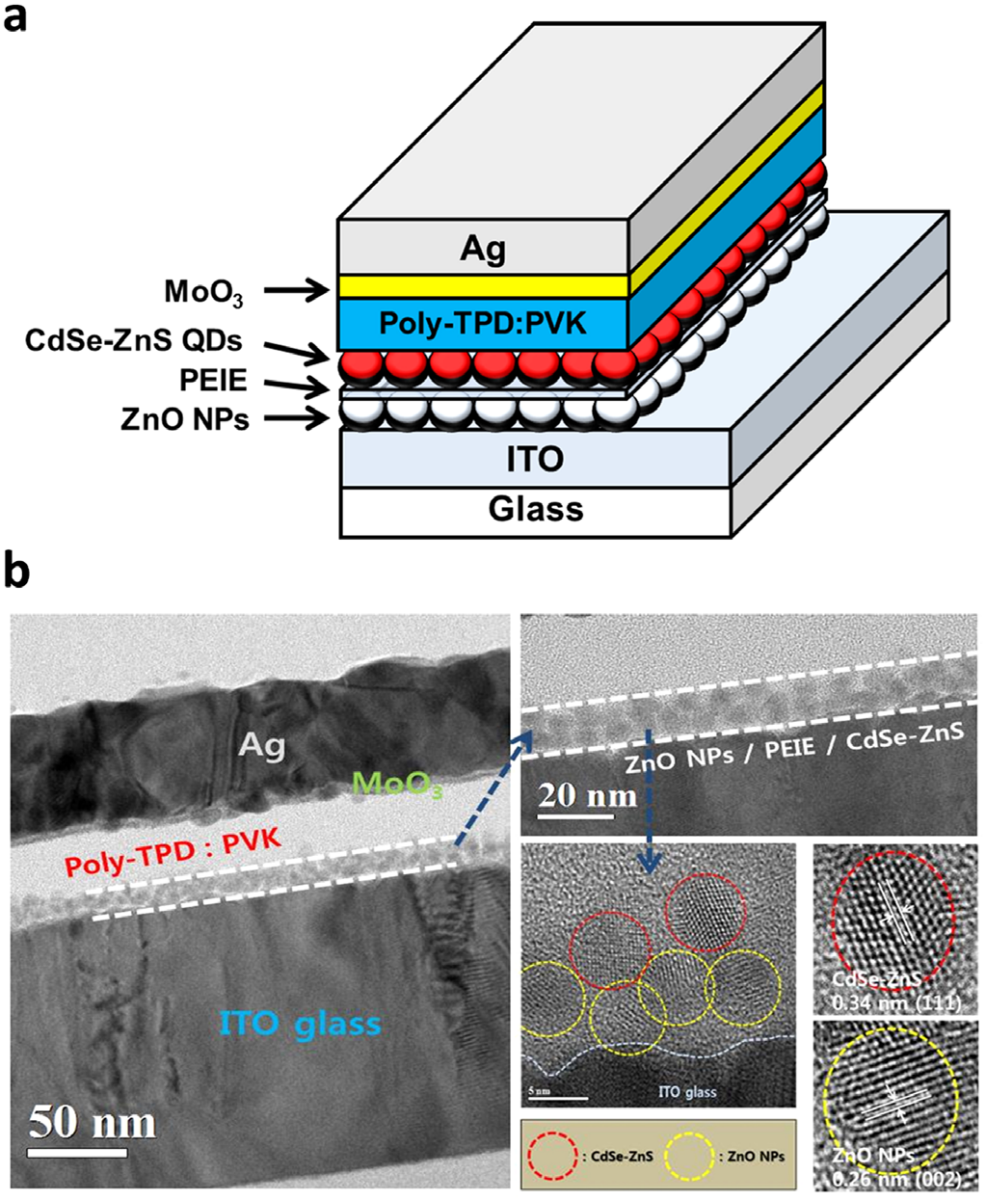
Device stucuture and TEM images. (a) A schematic cross-sectional view of inverted red QDLED and (b) corresponding cross-sectional high-resolution transmission electron microscopy (HRTEM) images.

A schematic cross-sectional view of inverted red QDLED and corresponding cross-sectional high-resolution transmission electron microscopy (HRTEM) images are shown in Fig. 1. The device consists of the patterned ITO (cathode), ZnO NPs/PEIE film as the EIL/ETL, CdSe-ZnS QDs as the emission layer (EML), the poly(N,N′-bis(4-butylphenyl)-N,N′-bis(phenyl)benzidine) (poly-TPD) and poly(N-vinylcarbazole) (PVK) blend layer as the hole transport layer (HTL), molybdenum trioxide (MoO_3_) as the hole injection layer (HIL), and Ag (anode). ZnO NPs were synthesized per procedures in the previously reported literature^17^ and other materials were commercially available. PEIE, ZnO NPs, CdSe-ZnS QDs, and poly-TPD:PVK layers were prepared by spin coating. In the cross-sectional TEM image (left side of Fig. 1b), boundaries of ZnO/PEIE/CdSe-ZnS QDs three layers were difficult to distinguish while those of Ag, MoO_3_, and poly-TPD:PVK layers were clear. In order to clarify boundaries, lattice fringes in magnified TEM images (right side) were carefully analyzed. ZnO NPs and CdSe-ZnS QDs have different crystal structures, resulting in clearly different lattice fringes as indicated by the red and yellow circles. CdSe-ZnS QDs has zinc blend structure and the interplanar distance of 0.34 nm corresponding to (111) plane was observed^20^. On the other hand, our synthesized ZnO NPs has wurtzite structure which was confirmed by the X-ray diffraction (XRD) pattern, although the small particle size (average diameter of 4–5 nm) led to a significant broadening of the characteristic diffraction (see Fig. S1a and S1b in the supplementary information)^16^. The interplanar distances of 0.26 nm between lattice fringes corresponded to the (002) plane of ZnO^8^. It is interesting to note that the PEIE layer in between ZnO NPs and CdSe-ZnS QDs is indistinguishable, which is attributed to infiltration by PEIE into ZnO NPs during solution process. However, the significant change in ultraviolet photoemission spectroscopy (UPS) spectra of ZnO NPs and ZnO NPs/PEIE films shows that PEIE had covered the top surface of ZnO NPs, as will be given below.

### Electronic energy level of ZnO NPs/PEIE

**Figure 2:**
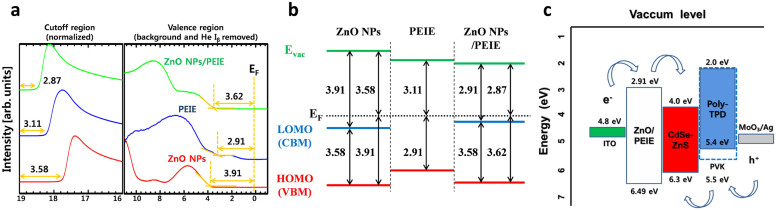
Electronic energy level. (a) Secondary cutoff region and valence (or HOMO) region after background removal obtained via UPS spectra of ZnO NPs, PEIE and ZnO/PEIE films deposited on ITO glass substrate, respectively, and (b) corresponding energy level diagrams. (c) Schematic illustration of energy band diagram of inverted QDLEDs consisting of ITO/ZnO NPs/PEIE/CdSe-ZnO QD/poly TPD:PVK/MoO_3_/Ag. The ZnO NPs/PEIE energy level was estimated from UPS and optical absorption measurement. Other energy level values are taken from literatures. The pathways of the holes and the electrons are indicated by the arrows.

Figure 2a and 2b displays the results of UPS spectra taken from ZnO NPs, PEIE, and ZnO NPs/PEIE spin coated on ITO and the corresponding energy level diagrams. The work functions could be calculated between the incident light energy (*hν* = 21.22 eV) and the energy (E_off_) of the secondary cutoff. In the case of ZnO NPs film on top of ITO, the WF is estimated to be 3.58 eV, which is much lower than that of a normal ZnO film (more than 4.0 eV)^21^. Valence band maximum (VBM) is measured to be 7.49 eV. Considering optical energy bandgap of 3.58 eV obtained from the absorption spectrum (please see Fig. S1c in the supplementary information), conduction band maximum (CBM) is calculated to be 3.91 eV, which is higher than the WF of 3.58 eV. The origin of the lowered WF of ZnO NPs is still unclear but could be correlated with the surface state of ZnO NPs. The photoluminescence (PL) spectrum shows a broad blue-green emission (see Fig. S1c in the supplementary information) that would be relevant to oxygen vacancies^7^. The synthesized 4–5 nm ZnO NPs have a very high surface area, indicating that many oxygen vacancies may exist in the surface. As the result, the oxygen-deficient surface of ZnO NPs leads to the significantly lowered WF which is consistent with the previously reported result from other study^7^. In the case of PEIE coated on ITO, the PEIE modifier substantially reduced the WF of ITO as low as 3.11 eV, consistent with our previously reported value^18^. PEIE also lowered energy levels of ZnO NPs, as shown by the measured values of WF at 2.87 eV, CBM at 2.91 eV, and VBM at 6.49 eV in the ZnO NPs/PEIE layer. Based on the above UPS results, the inverted QDLEDs adopting the ZnO NPs/PEIE layer would be most favorable for electron charge injection into the QD emission layer. The energy band diagram of the inverted QDLEDs consisting of ITO/ZnO NPs/PEIE/CdSe-ZnO QDs/poly- TPD:PVK/MoO_3_/Ag were illustrated as shown in Fig. 2c (note: except for ZnO NPs/PEIE, other energy level values are taken from literatures^22^,^23^).

### Red QDLED device performance

**Figure 3:**
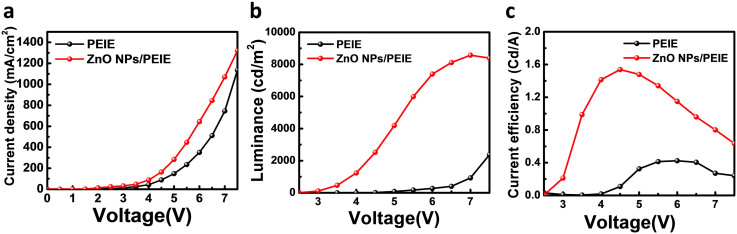
QDLED device characteristics. (a) Current density versus voltage, (b) luminance versus voltage, and (c) current efficiency versus voltage characteristics of QDLEDs with PEIE and ZnO NPs/PEIE layers.

Figure 3a shows the current density-voltage (*J*–*V*) characteristics of two inverted QDLED types, one adopting PEIE and another adopting ZnO NPs/PEIE layers. The QDLED with ZnO NPs/PEIE layer has a lower turn-on voltage of 2.0–2.5 V and the higher current density than the device with only PEIE single layer (3.0–3.5 V), which is consistent with the UPS results. The ZnO NPs/PEIE layer also improved luminance and current efficiency characteristics. As shown in Fig. 3b and 3c, the device with ZnO NPs/PEIE layer displays the maximum values of luminance of 8600 cd/m^2^ at 7 V and current efficiency of 1.53 cd/A at 4.5 V, which are at least three times higher than those of the device with PEIE single layer (2400 cd/m^2^ at 7.5 V and 0.42 cd/A at 6 V). These enhancements in luminance and current efficiency properties could be correlated with the improvement of charge balance in the QD emission layer, because the ZnO NPs functions not only as an electron transport layer but also as an efficient hole blocking layer. PEIE as surface modifier can modulate the WF of host material such as ITO or ZnO NPs to improve electron injection, but cannot play a role of a hole blocking layer by itself. Therefore, the use of ZnO NPs/PEIE layer facilitates the enhancement of both electron injection into the QD emissive layer and charge balance on the QD emitter, resulting in improvement of device performance.

### Full color QDLED demonstration

**Figure 4:**
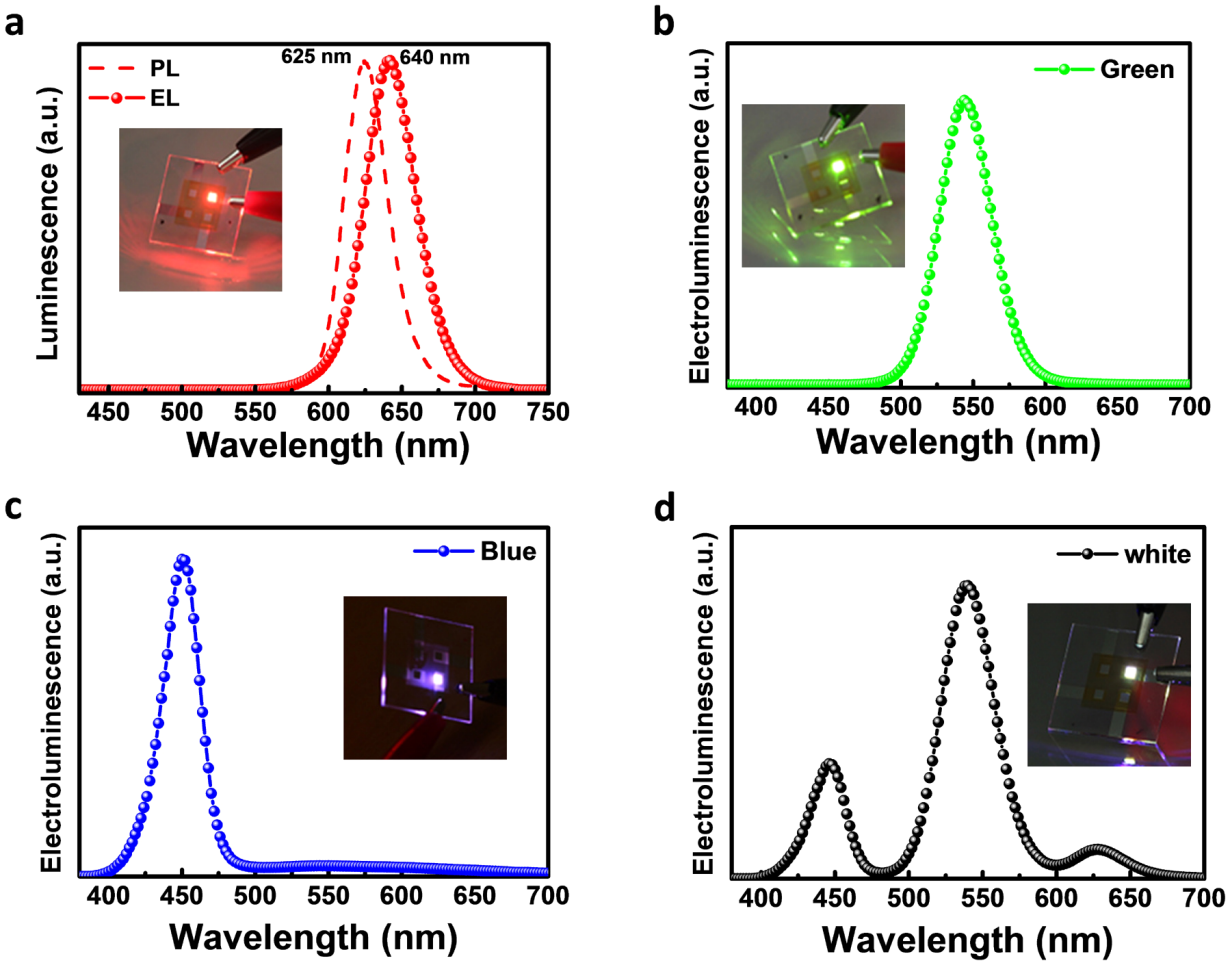
Red, Green, Blue, and White full color QDLED. (a) Photoluminescence spectrum of CdSe-ZnS QDs solution (dashed line) and electroluminescence spectra (solid line with circle symbol) of red QDLEDs adopting the ZnO NPs/PEIE layer. Electroluminescence spectra of (b) green, (c) blue, and (d) white QDLEDs with ZnO NPs/PEIE layers. Insets show photographs of red, green, blue, and white devices with ZnO NPs/PEIE layer at applied voltages of 6.5–7.5 V.

Figure 4a exhibits the PL spectrum of dilute red CdSe-ZnS QDs solution (dashed line) and electroluminescence (EL) of the device adopting the ZnO NPs/PEIE layer. The EL spectrum centered at 640 nm presents saturated emission from QD and is red-shifted by about 15 nm relative to the peak position of solution PL spectrum (625 nm). The redshift is attributed to the enhanced interdot interactions arising from the reduced interdot distance in close-packed CdSe-ZnS film and/or to the electric-field-induced Stark effect^7^. The inset of Fig. 4a is a photograph of the red light emission at 7 V with the Commission Internationale de l′Eclairage (CIE) color coordinate of (0.68, 0.31). The same scheme with ZnO NPs/PEIE layer can be applied in fabrication of full color inverted structure QDLEDs. We demonstrate green, blue and white inverted QDLEDs (note: for white color emission, red, green, and blue CdSe-ZnS QDs were mixed in a weight ratio of 1:3:10). The EL spectra and photographs of green, blue, and white QDLEDs were shown in Fig. 4b–4d. The devices reached the maximum values of luminance and current efficiency at 6.5–7.5 V: 18000 cd/m^2^ and 1.26 cd/A with the CIE coordinate of (0.26, 0.70) for the green QDLED, 150 cd/m^2^ and 0.006 cd/A with the CIE coordinate of (0.17, 0.6) for the blue QDLED, and 4500 cd/m^2^ and 0.48 cd/A with the CIE coordinate of (0.25, 0.46) for the white QDLED. Details on luminance-voltage and current efficiency-voltage characteristics of green, blue and white CdSe-ZnS QDs are reported in Fig. S2 in the supplementary information. As such, a simple addition of ZnO NPs layer to our PEIE-modified QDLED design has brought significant improvements to the device. The maximum device performance is yet to be realized as further optimization of the device design is still warranted.

In conclusion, we introduce a PEIE-modified ZnO NPs electron injection/transport layer for inverted red QDLED. The combination of PEIE and ZnO NPs facilitates the enhancement of both electron injection into the CdSe-ZnS QD emissive layer and charge balance on the emission layer. As a result, the device with the ZnO NPs/PEIE layer exhibits a low turn-on voltage of 2–2.5 V and maximum values of luminance of 8600 cd/m^2^ at 7 V and current efficiency of 1.53 cd/A at 4.5 V which are at least three times higher than those of the device with PEIE single layer (2400 cd/m^2^ at 7.5 V and 0.42 cd/A at 6 V). Furthermore, the same scheme with ZnO NPs/PEIE layer was applied in fabrication of full-color inverted structure QDLEDs and we demonstrate green, blue and white inverted QDLEDs. We thus conclude that the use of ZnO NPs/PEIE is a promising and practical approach to realization of high performance, easy-to-fabricate, large area inverted QDLEDs, paving the way for development of next generation displays and solid-state lighting.

## Methods

### Device fabrication of Inverted QDLED

Inverted structure red QDLED was fabricated on ITO glass substrates. Before device fabrication, the patterned ITO substrates were cleaned by sonication sequentially in acetone, methanol, and isopropyl alcohol. And then, the ITO substrates were rinsed with deionized water and treated with O_2_ plasma. ZnO NPs (20 mg/ml, in butanol) layer was deposited on the ITO glass by spin coating at 4000 rpm for 40 s and dried at 110°C for 30 min in a nitrogen glove box. PEIE was dissolved in H_2_O with a concentration of 35–40 wt.% when received from Aldrich. Then it was further diluted with 2-methoxyethanol to a weight concentration of 0.2 wt %. The solution was spin coated on top of the ZnO NPs layer at 6000 rpm for 40 s, followed by thermal annealing at 110°C for 30 min. The CdSe-ZnO QDs layer was then deposited on the ITO/ZnO NPs/PEIE layer by spin coating using the colloidal QD solution dispersed in toluene (20 mg/mL) at 4000 rpm for 40 s and then annealed at 130°C for 1 h in the nitrogen glove box. The blend of poly-TPD and PVK with a ratio of 1:1 in weight were dissolved in chlorobenzene and then deposited on the top of QD layer by spin coating at 4000 rpm for 40 s and annealed at 130°C to remove the residual solvent. Finally, MoO_3_ and Ag layers were sequentially patterned through a shadow mask by thermal evaporation.

### Synthesis of ZnO nanoparticle

ZnO NPs were synthesized per procedures in the previously reported literature^17^. Zinc acetate dehydrate (Zn(Ac)_2_·2H_2_O) is used as a starting material. A precursor solution was prepared as follow: 1.23 g of Zn(Ac)_2_·2H_2_O was dissolved in 55 ml of methanol at room temperature and 0.48 g of potassium hydroxide (KOH) was dissolved in 25 ml of methanol at 60°C. The two individual solutions were mixed and stirred at 60°C for 2 hours under nitrogen ambient. ZnO NPs as white precipitate appeared, which need to be further purified by centrifugation and washing with methanol. Finally, ZnO NPs were dispersed in butanol solvent with concentration of 20 mg/mL. ZnO NPs were confirmed by XRD, ultraviolet-visible (UV-Vis) and PL measurements. The ZnO NPs sizes were measured with TEM.

### Material and device characterization

TEM (Model: JEM 2100F) at the accelerationvoltage 200 kV was carried out to investigate the cross-sectional structure of the inverted QDLEDs, using focused ion beam (FIB) milling techniques. UPS and UV-Vis analyses were used to evaluate electronic structure. For the measurement of PL, a He–Cd laser (λ = 325 nm) was used as the excitation source. The current-voltage-luminance characteristics of the devices were measured with a Spectra Scan PR-670 spectroradiometer and a Keithley-2601 source-measure unit.

## Author Contributions

D.H., W.C., D.S. and C.P. organized this project. H.K and D.H. designed experiments and fabricated the devices. S.P. and Y.Y. preformed the UPS measurements and analyzed results. H.K., D.H. and W.C. wrote the draft of the manuscript and all authors contributed to the scientific interpretation as well as to the edits of the manuscript.

## Supplementary Material

Supplementary Information Supplementary Information
