# Supplementary material for: Inverted Quantum Dot Light Emitting Diodes using Polyethylenimine ethoxylated modified ZnO
Source: Sci Rep. 2015 Mar 10;5:8968. doi: 10.1038/srep08968 (PMC4354089; doi:10.1038/srep08968)
Supplement: Supplementary Information [file srep08968-s1.pdf]

Supplementary Information

Inverted Quantum Dot Light Emitting Diodes using Polyethylenimine ethoxylated modified ZnO

Hong Hee Kim[1,2], Soohyung Park[3], Yeonjin Yi[3], Dong Ick Son[4], Cheolmin Park[2], Do Kyung Hwang[1,5]★, and Won Kook Choi[1,5]★

[1]Interface Control Research Center

Future Convergence Research Division

Korea Institute of Science and Technology (KIST), Seoul 136-791, Korea

[2]Department of Materials Science and Engineering

Yonsei University, Seoul 120-749, Korea

[3]Institute of Physics and Applied Physics

Yonsei University, Seoul 120-749 (South Korea)

[4]Soft Innovative Materials Research Center

Korea Institute of Science and Technology (KIST), Jeonbuk 565-905, Korea

[5]Department of Nanomaterials and Nano Science

University of Science and Technology (UST), Daejun 305-350, Korea

★e-mail: dkhwang@kist.re.kr; wkchoi@kist.re.kr

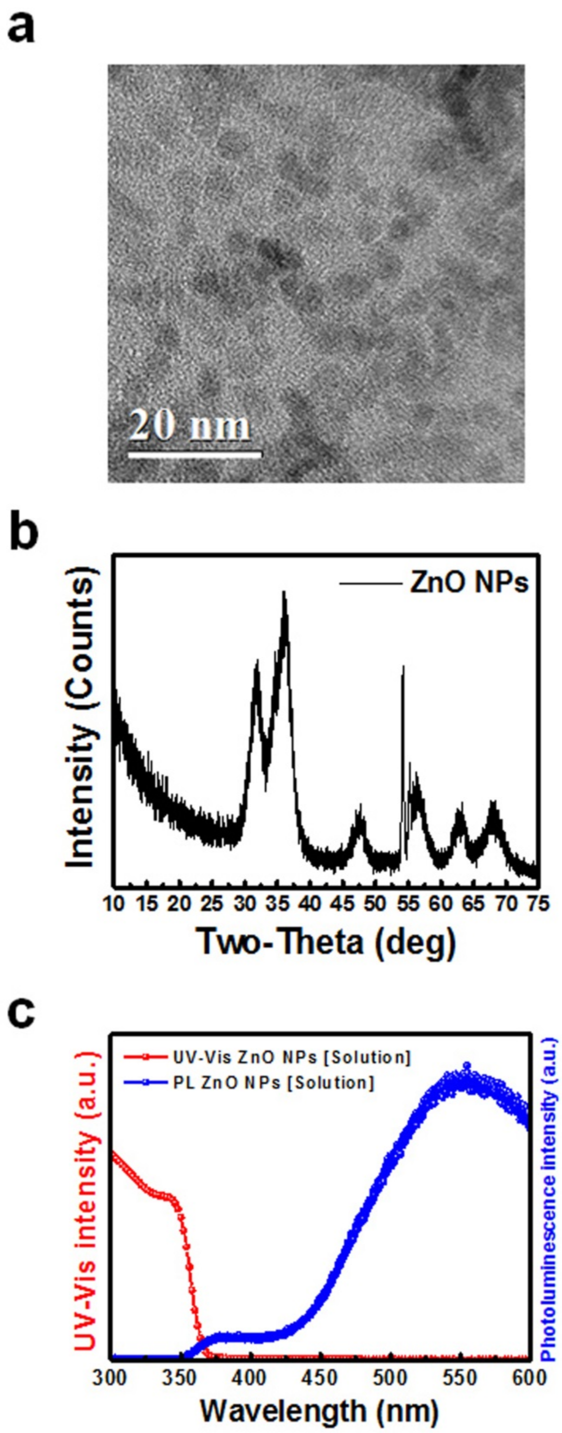

Figure S1. (a) XRD pattern from ZnO NPs film on Si substrate. (b) TEM image of ZnO NPs. (c) Absorption and photoluminescence spectra obtained from colloidal ZnO NPs solution dispersed in butanol. Excitation wavelength is 300 nm.

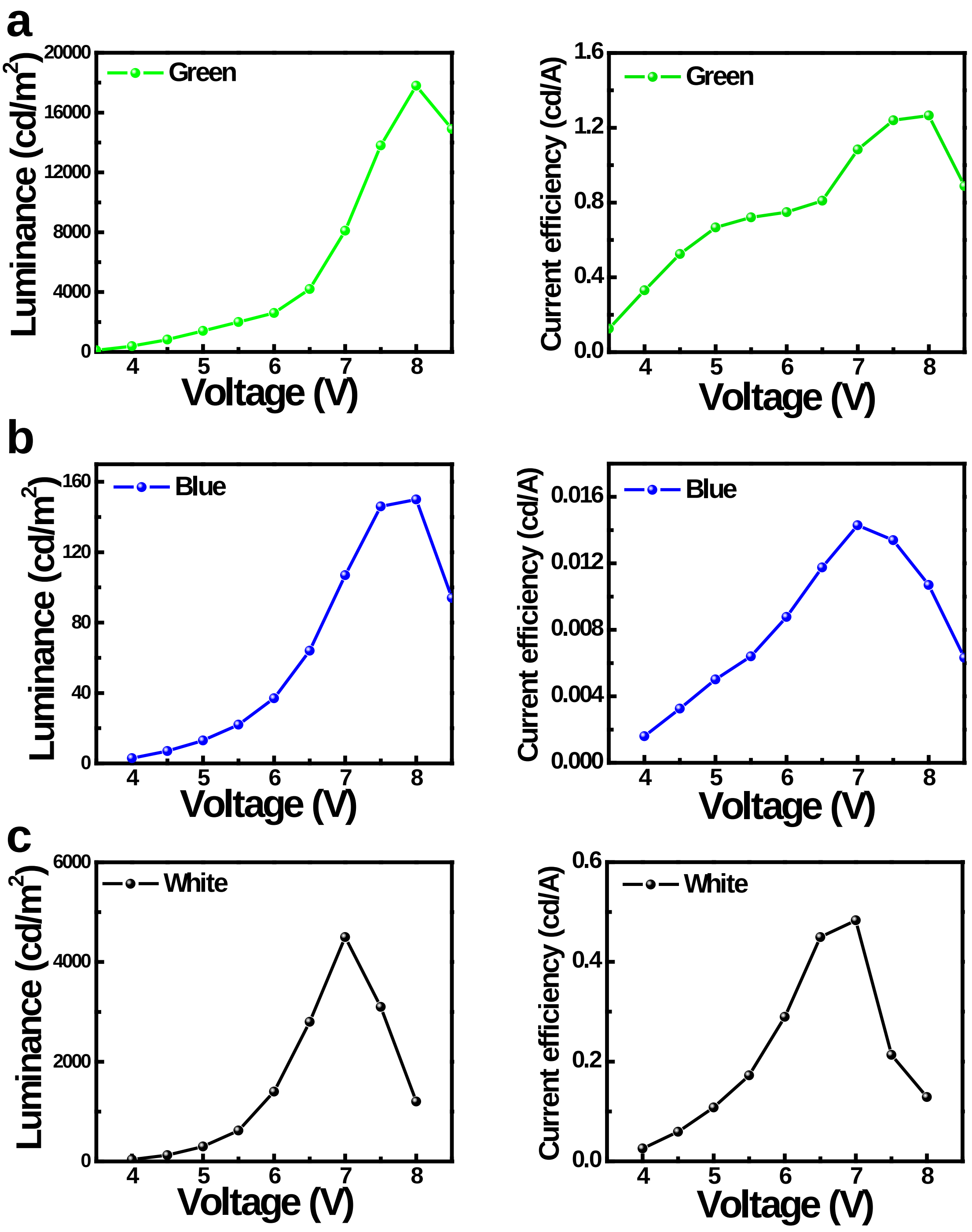

Figure S2. Luminance versus voltage and current efficiency versus voltage characteristics of (a) Green, (b) Blue, and (c) white QDLEDs.
